# Prognostic value of the TAPSE/PASP-ratio in patients with severe mitral regurgitation undergoing transcatheter edge-to-edge mitral valve repair

**DOI:** 10.1186/s12872-026-06180-2

**Published:** 2026-07-02

**Authors:** Felix Ausbuettel, Fares Kano, Nikolaos Patsalis, Christin Fichera, Dimitar Divchev, Carlo-Federico Fichera

**Affiliations:** 1 Department of Cardiology, University Hospital Marburg, Baldingerstraße, Marburg, 35043 Germany; 2 Faculty of Medicine, Justus-Liebig University Giessen, Ludwigstraße 23, Giessen, 35390 Germany; 3 University Hospital Greifswald, Clinic and Polyclinic for Internal Medicine B, Ferdinand-Sauerbruch-Straße, Greifswald, 17475 Germany; 4 Department of Cardiology, County Hospital Loerrach, Spitalstraße 25, Loerrach, 79539 Germany

**Keywords:** M-TEER, Right ventricular dysfunction, RV-PA uncoupling, MitraClip, PASCAL

## Abstract

**Background:**

Transcatheter edge-to-edge mitral valve repair (M-TEER) represents an effective treatment modality for high-grade mitral valve regurgitation. The right ventricle (RV) to pulmonary artery (PA) coupling ratio has been indicated as a marker of right ventricular dysfunction (RVD), but evidence among M-TEER patients remains inconsistent to date due to divergent definitions. We therefore aimed to shed light on the impact of RV-PA uncoupling on survival following M-TEER.

**Methods:**

Data from all patients who underwent M-TEER and provided sufficient echocardiographic data were investigated. RV-PA uncoupling was defined as the ratio of tricuspid annular pulse systolic excursion (TAPSE) and to the Doppler echocardiographic-derived pulmonary artery systolic pressure (D-PASP) < 0.37 mm/mmHg.

The difference in long-term survival between patients with and without RV-PA uncoupling were analyzed via the Kaplan-Meier method, and independent predictors of mortality were identified via uni- and multivariable Cox regression analyses.

**Results:**

A total of 158 patients were eligible for analysis, and RV-PA uncoupling was present in 32.3% of the patients (51/158). Patients with RV-PA uncoupling presented significantly advanced congestive heart failure stages. While M-TEER was performed equally safely in patients with RV-PA uncoupling (odds ratio for procedural success: 0.95, 95% confidence interval [CI] 0.29–2.77, *p* = 0.9), their long-term survival three years after M-TEER was significantly worse (50.9% (26/51) vs. 61.7% (66/107), *p* = 0.01). In this regard, a TAPSE/D-PASP ratio < 0.37 mm/mmHg proved to be a more consistent discriminator of long-term survival than a TAPSE < 18 mm alone.

**Conclusion:**

RV-PA uncoupling, defined as a TAPSE/D-PASP ratio of < 0.37 mm/mmHg, is a feasible and reproducible parameter, which also serves as a marker for advanced congestive heart failure and worse survival outcomes.

**Supplementary Information:**

The online version contains supplementary material available at 10.1186/s12872-026-06180-2.

## Background

Owing to ongoing demographic changes, mitral valve regurgitation (MR) represents a widespread valvular heart disease (VHD) among patients aged > 75 years [1]. Transcatheter edge-to-edge mitral valve repair (M-TEER) is gaining momentum as an efficacious treatment modality, since the majority of today’s patients suffering from congestive heart failure and high-grade MR are not eligible for cardiac surgery due to the increased perioperative mortality risk [2–5].

Preliminary data indicate a negative impact of right ventricular dysfunction (RVD) on long-term survival [6, 7].

Nevertheless, the quantification of RVD remains challenging because of the multitude of parameters and the dynamic right ventricular load-dependent morphology [8]. In this context, the right ventricle (RV)-to-pulmonary artery (PA) coupling ratio has been developed as a functional noninvasive parameter that has already been shown to be correlated with mortality and hospitalization rates among congestive heart failure patients [9].

However, consecutive evidence in M-TEER patients is hampered by the inconsistent threshold definition of RV-PA uncoupling between studies [10–15]. Due to the consistently growing number of patients, along with the subsequent burden on public health care systems, the most precise discrimination of long-term prognosis is necessary.

We therefore sought to investigate the prevalence of RV-PA uncoupling in a cohort of “real-world” patients who underwent M-TEER and its value as a discriminator of long-term survival.

## Methods

All patients who underwent M-TEER in this monocentric retrospective cohort study were enrolled for further analysis. The interdisciplinary heart team, consisting of interventional cardiologists and cardiac surgeons, determined that all patients were ineligible candidates for surgical mitral valve repair. Prior to the intervention, the morphological suitability for M-TEER was confirmed via transthoracic and transoesophageal echocardiography. For this purpose, the heart team followed the echocardiographic criteria outlined in the Endovascular Valve Edge-to-Edge Repair Study (EVEREST) II for patients with degenerative MR [4] and those of the Cardiovascular Outcomes Assessment of the MitraClip Percutaneous Therapy for Heart Failure Patients with Functional Mitral Regurgitation (COAPT) study for patients with functional MR [16]. Procedural success was defined as a reduction in MR to moderate severity or less in the absence of mitral valve stenosis with mean gradient ≥ 5 mmHg. Either the MitraClip© (Abbott Vascular, Chicago, IL, USA) or the PASCAL™ system (Edwards Lifesciences, Irvine, CA, USA) were utilized for the M-TEER procedure, the corresponding details regarding the implantation techniques have been published previously [5, 17, 18]. Procedure-associated complications, including major adverse cardiovascular and cerebrovascular events (MACCEs), were reported in a standardized manner following the Mitral Valve Academic Research Consortium (MVARC) classification [19].

At the time of index hospitalization, RV-PA coupling was estimated using the ratio of tricuspid annular systolic excursion (TAPSE) and Doppler echocardiographic-derived pulmonary artery systolic pressure (D-PASP).

RV-PA uncoupling was defined as a TAPSE/D-PASP ratio < 0.37 mm/mmHg in line with previous studies in M-TEER patients, which defined RV-PA uncoupling using a TAPSE/PASP ratio in the range of 0.27–0.37 mm/mmHg [11, 12, 14]. The eligible patients were subsequently allocated to the respective cohorts according to the degree of RV-PA uncoupling. Patients whose available echocardiographic recordings were not sufficient for retrospective calculation of the TAPSE/D-PASP ratio were excluded from further analysis.

The observation period of the study ranged from May 2014 to May 2023. The documented medical history, including ambulatory visits and telephone consultations in the context of the treatment, were used for the analysis of the follow-up period. The primary endpoint of the analysis was mortality during the follow-up period. The median duration of the follow-up period was 499 ± 832 days in the overall cohort, with a non-significant trend towards a reduced follow-up period in patients with RV-PA uncoupling compared to patients without RV-PA uncoupling (399 ± 805 days vs. 532 ± 758 days, *p* = 0.06). A loss to follow-up was defined as a follow-up period of less than 30 days. Despite intensified efforts, loss to follow-up was observed in 5.1% (8/158) of patients, with no significant difference in distribution between patients with and without RV-PA uncoupling (9.8% (5/51) vs. 2.8% (3/107), *p* = 0.1).

The study was implemented in accordance with the guidelines of the Declaration of Helsinki and was approved by the Ethics Committee of the Faculty of Medicine of Philipps University of Marburg prior to initiation (protocol code: RS 23–163, clinical trial number: not applicable). Informed consent was waived because of the retrospective nature of the study.

### Statistical analysis

All the statistical analyses were conducted using R Studio V4.4.2 (R Foundation for Statistical Computing, Vienna, Austria) and the “survival”, “survminer”, “dplyr”, “Rcmdr”, “TableOne” and “My.stepwise” packages.

The presented graphics were designed with Biorender.com (Science Suite Inc., Toronto, ON, Canada). Continuous variables are reported as the means with standard deviations (SDs) for normally distributed data and by medians and interquartile ranges (IQRs; 25th-75th percentiles) for nonnormally distrbuted data. The assumption of a normal distribution was validated using the Shapiro-Wilk test. Categorical variables are presented as absolute and relative frequencies (%). Differences between the compared groups were tested for statistical significance using the Student’s t-test for normally distributed continuous variables and Wilcoxon’s test for nonnormally distributed continuous variables. Likewise, the chi-squared test was used for categorical variables at expected cell sizes ≥ 20 and Fisher’s exact test at expected cell sizes < 20. To plot the course of long-term survival following M-TEER, the Kaplan-Meier method was applied, and subsequent differences were compared using the logrank test. Independent predictors of mortality were analyzed using uni- and multivariable Cox regression analysis, with those variables with a p-value of < 0.1 in the univariable analysis were included in the multivariable model. A two-sided p-value of ≤ 0.05 was considered statistically significant.

## Results

A total of 183 patients underwent M-TEER during the observation period. Owing to insufficient echocardiographic data, 25 patients were excluded from further analysis. A comparison of the clinical characteristics of the included and excluded patients is provided in Supplementary Table S1.

The remaining 158 patients were eligible for further analysis, and RV-PA uncoupling was present in 32.3% (51/158) of the patients.

### Clinical cohort characteristics

Compared with patients without RV-PA uncoupling, patients with confirmed RV-PA uncoupling presented advanced stages of congestive heart failure at the time of index hospitalization, leading to significantly higher values of the STS risk score and euroSCORE II, which are established scores for predicting periinterventional mortality [20]. No significant differences were observed with respect to the prevalence of functional MR etiology between patients with and without RV-PA uncoupling (66.7% (34/51) vs. 67.3% (72/107), *p* = 1). However, patients with RV-PA-uncoupling as a consecutive marker of right ventricular dysfunction and pulmonary hypertension had a higher rate of severe dyspnea, corresponding to Class IV of the New York Heart Association (NYHA) classification. In association with RV-PA uncoupling, a consecutive pattern of RVD, characterized by reduced TAPSE at elevated pulmonary artery pressures, was identified by echocardiography.

The remaining clinical characteristics were equally distributed between the cohorts and are summarized in Table 1.

**Table 1:** Clinical and procedural characteristics between patients with and without RV-PA uncoupling◊

Variable	Overall cohort (n=158)	RV-PA Coupling (n=107)	RV-PA Uncoupling (n=51)	p-value
Age (years)	79 ± 7	79 ± 7	80 ± 7	0.3
Male sex	59.5% (94)	57.9% (62)	62.7% (32)	0.7
BMI (kg/m²)	27 ± 5	27.4 ± 5	27 ± 4	0.3
euroSCORE II (%)*	7.2 ± 10.4	6.5 ± 6.7	12 ± 24.6	0.001
STS-Risk-Score (%)*	6.1 ± 5.9	5.4 ± 4.3	8.9 ± 11.8	<0.001
MitraScore	3.6 ± 1.4	3.5 ± 1.5	3.7 ± 1.4	0.4
Procedure duration (min)	96 ± 39	95 ± 38	98 ± 40	0.7
Number of implanted clips	1.3 ± 0.5	1.3 ± 0.5	1.2 ± 0.5	0.6
COPD	24.7% (39)	24.3% (26)	25.5% (13)	1
CAD	73.4% (116)	72.9% (78)	72.5% (37)	1
Pacemaker	25.9% (41)	31.4% (27)	27.5% (14)	0.3
Prior CRT	16.5% (26)	14% (15)	17.7% (9)	0.5
+ ICD	20.3% (32)	21.5% (23)	17.7% (9)	0.7
Diabetes mellitus	33.5% (53)	32.7% (35)	35.3% (18)	0.9
Pulmonary hypertension	53.2% (84)	59.8% (64)	39.2% (20)	0.8
Precapillary PH	2.5% (4)	2.8% (3)	1.9% (1)	
Ipc-PH	22.2% (35)	27.1% (29)	11.8% (6)	0.5
Cpc-PH	28.5% (45)	29.9% (32)	25.5% (13)	
Arterial hypertension	88.6% (140)	86% (92)	94.1% (48)	0.2
Prior CAB-OP	18.4% (29)	14% (15)	27.5% (14)	0.07
Prior PCI	57.6% (91)	61.7% (66)	49% (25)	0.2
Previous Stroke	12% (19)	10.3% (11)	15.7% (8)	0.5
Atrial fibrillation	73.4% (116)	72.9% (78)	74.5% (38)	1
PAD	12.7% (20)	12.1% (13)	13.7% (7)	1
NYHA III	58.2% (92)	66.4% (71)	41.2% (21)	0.02
NYHA IV	38% (60)	29.9% (32)	54.9% (28)	
NTproBNP (pg/mL)*	1426 ± 3182	1253 ± 3186	1505 ± 2893	0.4
GFR (mL/min)	47 ± 21	49 ± 20	43 ± 22	0.09
Length of postinterventional hospital stay (d)*	7 ± 4	7 ± 3	7 ± 4	0.6
Heart failure therapy				
Beta blockers	82.2% (130)	85% (91)	76.5% (39)	0.3
ACE inhibitors/AT1 blockers	72.8% (115)	72% (77)	74.5% (38)	0.9
ARNI	7.6% (12)	8.4% (9)	5.9% (3)	0.8
No RAAS-inhibitor therapy	20.3% (32)	20.6% (22)	19.6% (10)	1
MRA	50.6% (80)	55.1% (59)	41.2% (21)	0.1
SGLT-II-inhibitors	13.3% (21)	13.1% (14)	13.7% (7)	1
Vericiguat	0.6% (1)	0.9% (1)	0% (0)	1
Diuretics	89.2% (141)	91.5% (96)	91.8% (45)	0.2
High dose Diuretics^#^	44.3% (70)	44.8% (48)	43.2% (22)	0.8
Echocardiographic parameters				
Periinterventional MR reduction (carpentier grade)	∆2.1 ± 0.5	∆2.1 ± 0.6	∆2.0 ± 0.5	0.3
TR grade III	22.2% (35)	17.8% (19)	31.4% (16)	0.09
LVEF (%)	45 ± 13	45 ± 12	44 ± 13	0.5
Transmitral gradient after M-TEER (mmHg)	3.3 ± 1.5	3.3 ± 1.4	3.3 ± 1.8	0.9
Functional MR etiology	67.1% (106)	67.3% (72)	66.7% (34)	1
LA diameter (mm)	47 ± 10	47 ± 10	47 ± 9	1
LVEDD (mm)	56 ± 9	56 ± 9	56 ± 9	1
TAPSE (mm)	18 ± 4	19 ± 4	15 ± 4	<0.001
D-PASP (mmHg)	41 ± 12	37 ± 10	49 ± 12	<0.001
Invasive parameters				
Right heart catheterization prior toM-TEER	56.3% (89)	61.7% (66)	45.1% (23)	0.06
RHC-PASP (mmHg)	58 ± 17	57 ± 15	61 ± 21	0.3
PVR (WU)	2.7 ± 1.8	2.7 ± 1.8	2.7 ± 1.6	0.9
PCWP (mmHg)	29 ± 9	29 ± 9	29 ± 9	0.8
Cardiac output (L/min)	4.2 ± 1.3	4.3 ± 1.4	4 ± 1.2	0.3

*ACE* Angiotensin-converting-enzyme, *ARNI* Angiotensin-receptor-neprilysin-inhibitor, *BMI* Body-Mass-Index, *CAB-OP* Coronary artery bypass-OP, *CAD* Coronary artery disease, *COPD* Chronic obstructive pulmonary disease, *Cpc-PH* Combined post- and precapillary pulmonary hypertension, *CRT* Cardiac resynchronization therapy, *D-PASP* Doppler-echocardiography measured pulmonary artery systolic pressure, *GFR* Glomerular filtration rate, *ICD* Implantable cardioverter defibrillator, *Ipc-PH* Isolated postcapillary pulmonary hypertension, *LA* Left atrium, *LVEDD* Left ventricular enddiastolic diameter, *LVEF* Left ventricular ejection fraction, *MR* Mitral valve regurgitation, *MRA* Mineralocorticoid receptor antagonist, *M-TEER* Transcatheter edge-to-edge mitral valve repair, *NYHA* New-York-Heart-Association, *PA* Pulmonary artery, *PAD* Periphereal arterial disease, *PCI* Percutaneous coronary intervention, *PCWP* Pulmonary capillary wedge pressure, *PH* Pulmonary hypertension, *PVR* Pulmonary vascular resistance, *RAAS* Renin-angiotensin-aldosterone system, *RHC-PASP* Right heart catheterization measured pulmonary artery systolic pressure, *RV* Right ventricle, *TAPSE* Tricuspid annular pulse systolic excursion, *TR* Tricuspid valve regurgitation

*Data presented as Median + IQR

^◊^Defined as TAPSE/D-PASP-ratio <0.37 mm/mmHg

^#^Requirement for intravenous diuretic therapy or furosemide equivalent dose >80mg/d

### Short-term outcomes

There was no association between RV-PA uncoupling and procedural success (odds ratio [OR] 0.95, 95% confidence interval [CI] 0.29–2.77, *p* = 0.9). Periinterventional hemorrhagic events were the most common complications overall, but there were no significant differences between the cohorts. An overview of the complication rates including MACCE rates is provided in Table 2.

**Table 2:** Short term complications including major adversive cardiovascular and cerebrovascular events (MACCEs) between patients with and without RV-PA uncoupling^◊^ after M-TEER intervention

Variable	Overall cohort (*n* = 158)	RV-PA Coupling (*n* = 107)	RV-PA Uncoupling (*n* = 51)	*p*-value
Thromboembolic event	1.3% (2)	1.1% (1)	2.2% (1)	1
Myocardial infarction	0% (0)	0% (0)	0% (0)	1
Bleeding	8.9% (14)	7.5% (8)	11.8% (6)	0.8
MVARC I	0% (0)	0% (0)	0% (0)	
MVARC II	3.2% (5)	1.9% (2)	6% (3)	
MVARC III	4.4% (7)	3.8% (4)	6% (3)	0.4
MVARC IV	0% (0)	0% (0)	0% (0)	
MVARC V	1.3% (2)	1.9% (2)	0% (0)	
Cardiac conduction system disturbances	0% (0)	0% (0)	0% (0)	1
In-hospital mortality	3.2% (5)	1.9% (2)	5.9% (3)	0.3
Cardiac cause	2.6% (4)	0.95% (1)	5.9% (3)	0.1
Non-cardiac cause	0.6% (1)	0.95% (1)	0% (0)	0.9

*D-PASP *Doppler-echocardiography measured pulmonary artery systolic pressure*. MACCEs  *Major cardiovascular and cerebrovascular events, *M-TEER *Transcatheter edge-to-edge mitral valve repair,* MVARC *Mitral valve academic research consortium* TAPSE *Tricuspid annular pulse systolic excursion

^**◊**^Defined as TAPSE/D-PASP-ratio < 0.37 mm/mmHg

### Long-term survival

Patients with concomitant RV-PA uncoupling had significantly worse long-term survival three years after M-TEER than did patients without RV-PA uncoupling (50.9% (26/51) vs. 61.7% (66/107), *p* = 0.01). Moreover, RV-PA uncoupling emerged as an independent predictor of mortality in the univariable Cox regression analysis (hazard ratio [HR] 2.0, 95%-CI 1.2–3.5, *p* = 0.01). However, in the multivariable analysis, male sex (HR 3.0, 95%-CI 1.2–7.3, *p* = 0.02) and concomitant high-grade tricuspid valve regurgitation (HR 2.6, 95%-CI 1.2–5.4, *p* = 0.01) emerged as significant predictors of mortality.

Nevertheless, RV-PA uncoupling emerged as a more appropriate discriminator of long-term survival than TAPSE alone, as patients with a measured reduction in TAPSE did not have significantly reduced survival compared with patients with normal TAPSE.

**Fig. 1:**
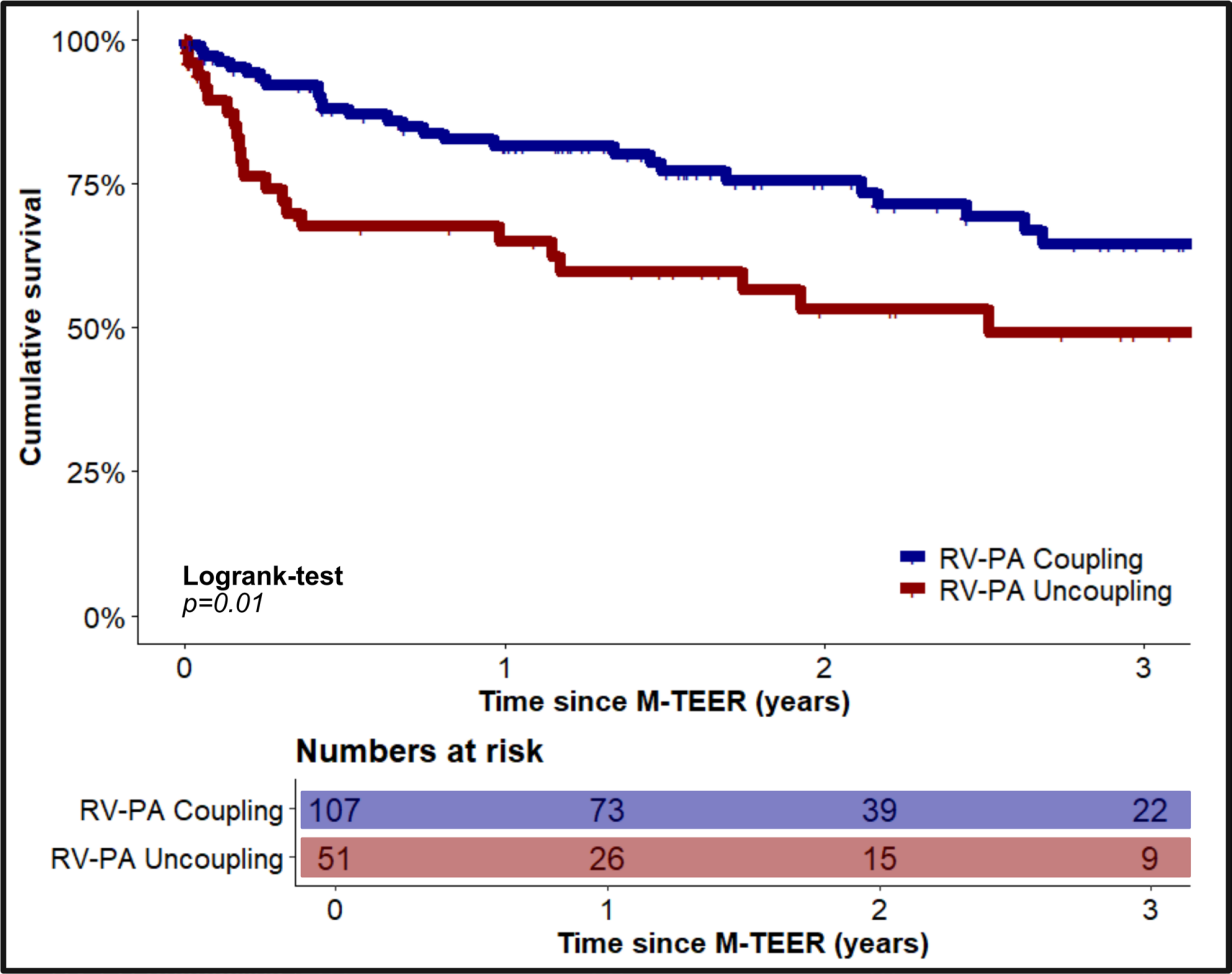
Long-term survival between patients with and without concomitant RV-PA uncoupling^◊^ after successful M-TEER. ◊Defined as TAPSE/D-PASP-ratio <0.37 mm/mmHg D-PASP – doppler-echocardiography measured pulmonary artery systolic pressure. M-TEER – transcatheter edge-to-edge mitral valve repair. PA – pulmonary artery. RV – right ventricle. TAPSE – tricuspid annular pulse systolic excursion

The course of long-term survival of the respective cohorts discriminated by RV-PA uncoupling is illustrated in Fig. 1, and that with the chosen reduction in TAPSE as a discriminator is shown in Supplementary Fig. 1. The significant predictors of mortality after uni- and multivariable Cox regression analyses are reported in Supplementary Table S2 and Table 3, respectively.

**Table 3:** Independent predictors of mortality after multivariable Cox regression analysis

Variable	Hazard ratio	95%-Confidence interval	*p*-value
Male sex	3.0	1.2–7.3	**0.02**
TR grade III	2.6	1.2–5.4	**0.01**

*TR *Tricuspid valve regurgitation

## Discussion

Despite remarkable therapeutic advancements in the interventional treatment of VHD over several decades, the overall prognosis of VHD aggravated by RVD remains poor [21, 22]. Our study enriches current evidence by providing an in-depth analysis of the impact of RV-PA uncoupling on long-term survival in a “real-world” cohort of patients who underwent M-TEER.

Concomitant RV-PA uncoupling was present in 32.3% (51/158) of the patients in this study. Despite the considerable scientific interest in RV-PA uncoupling as a marker of right ventricular dysfunction, no standardized threshold value has yet been determined in published studies. While some studies have suggested a threshold value for the TAPSE/PASP ratio of < 0.37 mm/mmHg [12, 14], another study used a threshold of < 0.27 mm/mmHg [11]. Further studies evaluated changes in the TAPSE/PASP ratio during the M-TEER procedure as a predictor of clinical outcomes without a definition of exact threshold values [10, 13, 15].

Consequently, further studies are needed to estimate the comparatively best threshold value for the TAPSE/PASP ratio. In this respect, a meta-analysis by Rmilah et al. was already able to identify a threshold value of < 0.3–0.37 mm/mmHg as a significant predictor of mortality and hospitalization following M-TEER [23].

The previously reported prevalence of RV-PA uncoupling among M-TEER patients varied between 25.8% and 64% [10–12, 14], although a comparison remains challenging due to the divergent definitions across the various studies. In general, the clinical characteristics of the present overall cohort were comparable to those of previously published clinical trials [10, 11, 14] and registries [17, 24].

In the present cohort, RV-PA uncoupling proved to be a marker of advanced symptomatic congestive heart failure with a consecutive increase in the risk of periinterventional mortality, as estimated via the euroSCORE II and STS risk score. These results were also reported by Karam et al. [11] and Koschutnik et al. [14].

In both studies, RV-PA uncoupling was also associated with a higher prevalence of cardiac comorbidities, which in principle could also have influenced the outcomes. A significant difference in cardiac comorbidities was not observed in the present cohort, so bias in the survival time analysis is unlikely.

Despite the increased predicted mortality risk, our study was able to demonstrate that M-TEER could be performed as safely in patients with RV-PA uncoupling as in patients without RV-PA uncoupling without significant differences in short-term mortality after M-TEER. Comprehensive data from other cohorts have not yet been reported regarding this aspect.

With respect to long-term outcomes, a significantly worse survival rate was observed among patients with concomitant RV-PA uncoupling than among patients without RV-PA uncoupling, which is in line with data from previous studies and registries [11, 12, 14, 25]. In this context, the shorter follow-up period – albeit not statistically significant – among patients with RV-PA uncoupling could be attributed to the higher mortality rate.

Pertaining to long-term survival, the selected cut-off value of < 0.37 mm/mmHg was validated as an appropriate discriminator. Furthermore, the definition of RV-PA uncoupling on the basis of the ratio of TAPSE to D-PASP proved to be effective owing to the simplified approach of measurement via conventional echocardiography, particularly in view of the complexity and variation of preload-dependent RV morphology, which subsequently impedes the calculation of functional RV parameters. In this study, the prediction of survival based on RV-PA uncoupling outperformed that based on TAPSE measurement alone, which, in principle, also represents a feasible and consistent assessment method. Nevertheless, after stratification of the cohort on the basis of the RVD as measured by TAPSE, no significant difference in long-term survival was noted. This finding contradicts the studies by Modin et al. [26] and Giovanardi et al. [27] who reported that TAPSE significantly predicted long-term survival in patients from the general community. However, there are fundamental differences in the clinical characteristics in relation to the present study cohort, as patients in the present study were significantly older, and burdened by significantly more complicating comorbidities, particularly symptomatic VHD. Moreover, RV-PA uncoupling permits a more comprehensive hemodynamic assessment of RVD than does the measurement of basal RV contractility using TAPSE alone. Although an independent influence of RV-PA uncoupling on mortality was identified by Kaplan-Meier analysis and univariable Cox regression analysis, the impact was not verified in the multivariable Cox regression analysis. This is most likely due to the rather small sample size from the single-center study design. Additionally, coexistent high-grade tricuspid regurgitation proved to be a strong predictor of mortality, in line with previous M-TEER studies [28, 29]. With respect to the underlying RVD, a coincidence between both variables could be assumed, although this was not confirmed statistically in the present study (OR 0.19, 95%-CI 0.02–1.64, *p* = 0.2).

In view of the available data, the overall question arises as to what extent the concept of M-TEER represents a reasonable therapeutic approach in patients with RV-PA uncoupling, as these patients presented at an advanced stage of VHD and had a significantly poorer survival rate. In this context, a study by Adamo et al. identified so-called responders whose RV-PA uncoupling improved in the course of M-TEER, which was associated with favourable outcomes compared with nonresponders [10]. Interestingly, an improvement in RV-PA uncoupling was observed in the majority of the cohort after receiving M-TEER, including patients with a comparatively low TAPSE/D-PASP ratio. Unfortunately, a comparative analysis cannot be performed in the present cohort due to insufficient data during the follow-up period. In terms of appropriate selection and referral of eligible patients for M-TEER intervention, the prediction of improvement for RV-PA uncoupling after M-TEER may also become a promising approach that should be further investigated in future studies.

### Limitations

Owing to the nature of an observational cohort study, no conclusions can be drawn with regard to any causalities. The single-center analysis limits the transferability of the results to other M-TEER cohorts. A more detailed analysis of right ventricular function via fractional area change and three-dimensional right ventricular ejection fraction would have been desirable; however, this analysis was not possible with the available data.

Furthermore, the influence of operator-related variations in the measurement of echocardiographic parameters on the results cannot be neglected in principle. Although only a small proportion of patients had to be excluded due to insufficient echocardiographic data, a bias in the results cannot be completely ruled out. The same applies to bias in the mortality analysis owing to unknown and uncollected cofactors. Nevertheless, highly relevant endpoints in clinical practice were addressed.

## Conclusion

RV-PA uncoupling was present in 32.3% (51/158) of current “real-world” M-TEER patients and served as an indicator of advanced VHD at the time of admission. Compared with patients without RV-PA uncoupling, patients with RV-PA uncoupling had significantly worse long-term survival. Baseline RV-PA uncoupling, defined as TAPSE/D-PASP ratio < 0.37 mm/mmHg, was a better discriminator of prognosis for survival than a baseline TAPSE < 18 mm. The existing evidence concerning the improvement of RV-PA uncoupling in the course of the M-TEER procedure should be further explored in prospective studies.

## Supplementary Information

Supplementary Material 1.

Supplementary Material 2.

## Data Availability

All data supporting the findings of this study are available within the paper and its Supplementary Information.

## Abbreviations

CI: Confidence interval
COAPT: Cardiovascular Outcomes Assessment of the MitraClip Percutaneous Therapy for Heart Failure Patients with Functional Mitral Regurgitation study
D–PASP: Doppler echocardiographic–derived pulmonary artery systolic pressure
EVEREST–II: Endovascular Valve Edge–to–Edge Repair Study
HR: Hazard ratio
IQR: Interquartile range
MACCE: Major adversive cardiovascular and cerebrovascular event
MR: Mitral regurgitation
MVARC: Mitral Valve Academic Research Consortium
M–TEER: Transcatheter edge–to–edge mitral valve repair
OR: Odds ratio
PA: Pulmonary artery
RV: Right ventricle
RVD: Right ventricular dysfunction
SD: Standard deviation
TAPSE: Tricuspid annular pulse systolic excursion
VHD: Valvular heart disease
