# Supplementary figures and images for: Prognostic value of the TAPSE/PASP-ratio in patients with severe mitral regurgitation undergoing transcatheter edge-to-edge mitral valve repair
Source: BMC Cardiovasc Disord. 2026 Jul 2;26:556. doi: 10.1186/s12872-026-06180-2 (PMC13326453; doi:10.1186/s12872-026-06180-2)

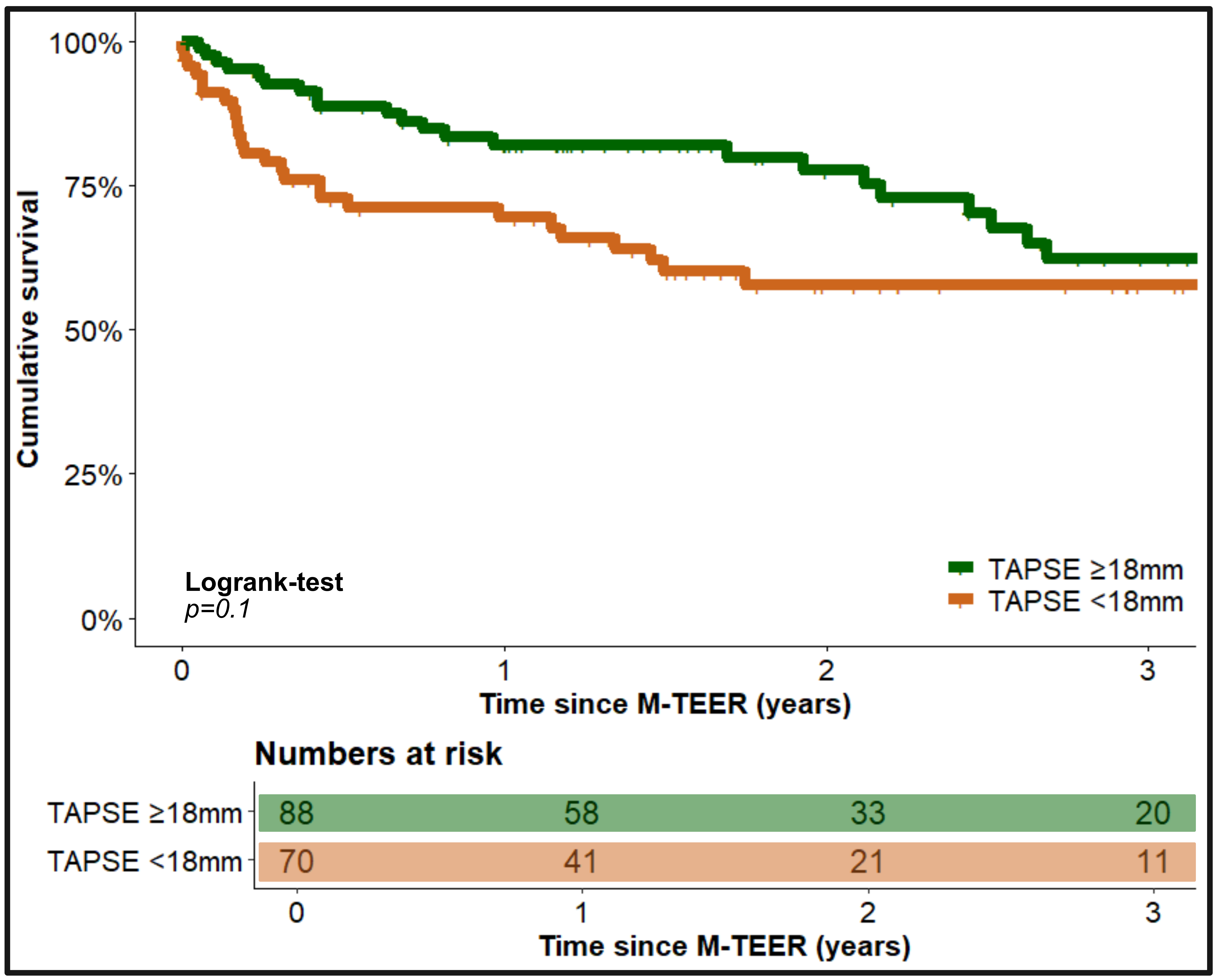

Supplement: Supplementary file 1 — Supplementary Material 1. [file 12872_2026_6180_MOESM1_ESM.png]
