# Supplementary material for: Prognostic value of the TAPSE/PASP-ratio in patients with severe mitral regurgitation undergoing transcatheter edge-to-edge mitral valve repair
Source: BMC Cardiovasc Disord. 2026 Jul 2;26:556. doi: 10.1186/s12872-026-06180-2 (PMC13326453; doi:10.1186/s12872-026-06180-2)
Supplement: Supplementary file 2 — Supplementary Material 2. [file 12872_2026_6180_MOESM2_ESM.docx]

**Supplementary Table S1: Clinical and procedural characteristics of patients with versus without sufficient echocardiographic recordings, which were then included or excluded from the study as appropriate**

| Variable | Included patients  (n=158) | Excluded patients  (n=25) | p-value |
| --- | --- | --- | --- |
| Age (years) | 79 ± 7 | 80 ± 6 | 0.4 |
| Male sex | 59.5% (94) | 44% (11) | 0.2 |
| BMI (kg/m²) | 27 ± 5 | 27 ± 5 | 0.8 |
| euroSCORE II (%)* | 7.2 ± 10.4 | 13.7 ± 18.4 | **0.02** |
| STS-Risk-Score (%)* | 6.1 ± 5.9 | 8.7 ± 15.5 | **0.03** |
| MitraScore | 3.6 ± 1.4 | 3.8 ± 1.4 | 0.4 |
| Procedure duration (min) | 96 ± 39 | 92 ± 31 | 0.6 |
| Number of implanted clips | 1.3 ± 0.5 | 1.3 ± 0.5 | 0.9 |
| COPD | 24.7% (39) | 20% (5) | 0.8 |
| CAD | 73.4% (116) | 80% (20) | 0.6 |
| Pacemaker  *Prior CRT*  *+ ICD* | 25.9% (41)  *16.5% (26)*  *20.3% (32)* | 88% (22)  *20% (5)*  *20% (5)* | **0.01**  *0.8*  *0.6* |
| Diabetes mellitus | 33.5% (53) | 36% (9) | 1 |
| Pulmonary hypertension | 53.2% (84) | 68.4% (13) | 0.5 |
| Arterial hypertension | 88.6% (140) | 88% (22) | 1 |
| Prior CAB-OP | 18.4% (29) | 44% (11) | **0.009** |
| Prior PCI | 57.6% (91) | 60% (15) | 1 |
| Previous Stroke | 12% (19) | 8% (2) | 0.8 |
| Atrial fibrillation | 73.4% (116) | 64% (16) | 0.5 |
| PAD | 12.7% (20) | 20% (5) | 0.5 |
| NYHA III NYHA IV | 58.2% (92)  38% (60) | 48% (12)  44% (11) | 0.6 |
| NTproBNP (pg/mL)* | 1426 ± 3182 | 1850 ± 2655 | 0.9 |
| GFR (mL/min) | 47 ± 21 | 43 ± 18 | 0.4 |
| Length of postinterventional hospital stay (d)* | 7 ± 4 | 7 ± 4 | 0.7 |
| Heart failure therapy |  |  |  |
| Beta blockers | 82.2% (130) | 72% (18) | 0.3 |
| ACE inhibitors/AT1 blockers | 72.8% (115) | 72% (18) | 1 |
| ARNI | 7.6% (12) | 4% (1) | 0.8 |
| No RAAS-inhibitor therapy | 20.3% (32) | 24% (6) | 0.9 |
| MRA | 50.6% (80) | 40% (10) | 0.4 |
| SGLT-II-inhibitors | 13.3% (21) | 12% (3) | 1 |
| Vericiguat | 0.6% (1) | 0% (0) | 1 |
| Diuretics | 89.2% (141) | 92% (24) | 0.9 |
| High dose Diuretics | 44.3% (70) | 56% (14) | 0.4 |

**Data presented as Median + IQR*

*^#^Requirement for intravenous diuretic therapy or furosemide equivalent dose >80mg/d*

*ACE – angiotensin-converting-enzyme. ARNI – angiotensin-receptor-neprilysin-inhibitor. BMI – Body-Mass-Index.*

*CAB-OP – coronary artery bypass-OP. CAD – coronary artery disease. COPD – chronic obstructive pulmonary disease.*

*CRT – cardiac resynchronization therapy. GFR – glomerular filtration rate. ICD – implantable cardioverter defibrillator.*

*MRA – mineralocorticoid receptor antagonist. M-TEER – transcatheter edge-to-edge mitral valve repair. NYHA – New-York-Heart-Association. PA – pulmonary artery. PAD – periphereal arterial disease. PCI – percutaneous coronary intervention. RV – right ventricle.*

*RAAS – renin-angiotensin-aldosterone system. TR – tricuspid valve regurgitation.*

**Supplementary Table S2: Independent predictors of mortality after univariable Cox regression analysis**

| Variable | Hazard ratio | 95%-Confidence interval | p-value |
| --- | --- | --- | --- |
| Male sex | 3.0 | 1.6-5.8 | **<0.001** |
| COPD | 2.0 | 1.1-3.4 | **0.02** |
| ICD | 1.5 | 1.02-2.3 | **0.03** |
| PAD | 2.4 | 1.3-4.6 | **0.007** |
| NYHA class IV | 1.9 | 1.1-3.2 | **0.02** |
| TR grade III | 3.7 | 2.1-6.5 | **<0.001** |
| RV-PA uncoupling^◊^ | 2.0 | 1.2-3.5 | **0.01** |
| PH | 3.0 | 0.9-9.8 | 0.07 |

**^◊^***Defined as TAPSE/D-PASP-ratio <0.37 mm/mmHg*

*COPD – chronic obstructive pulmonary artery disease. NYHA – New-York-Heart-Association.*

*ICD – implantable cardioverter defibrillator. PA – pulmonary artery. PAD – peripheral artery disease.*

*PH – pulmonary hypertension. RV – right ventricle. TR – tricuspid valve regurgitation*

**
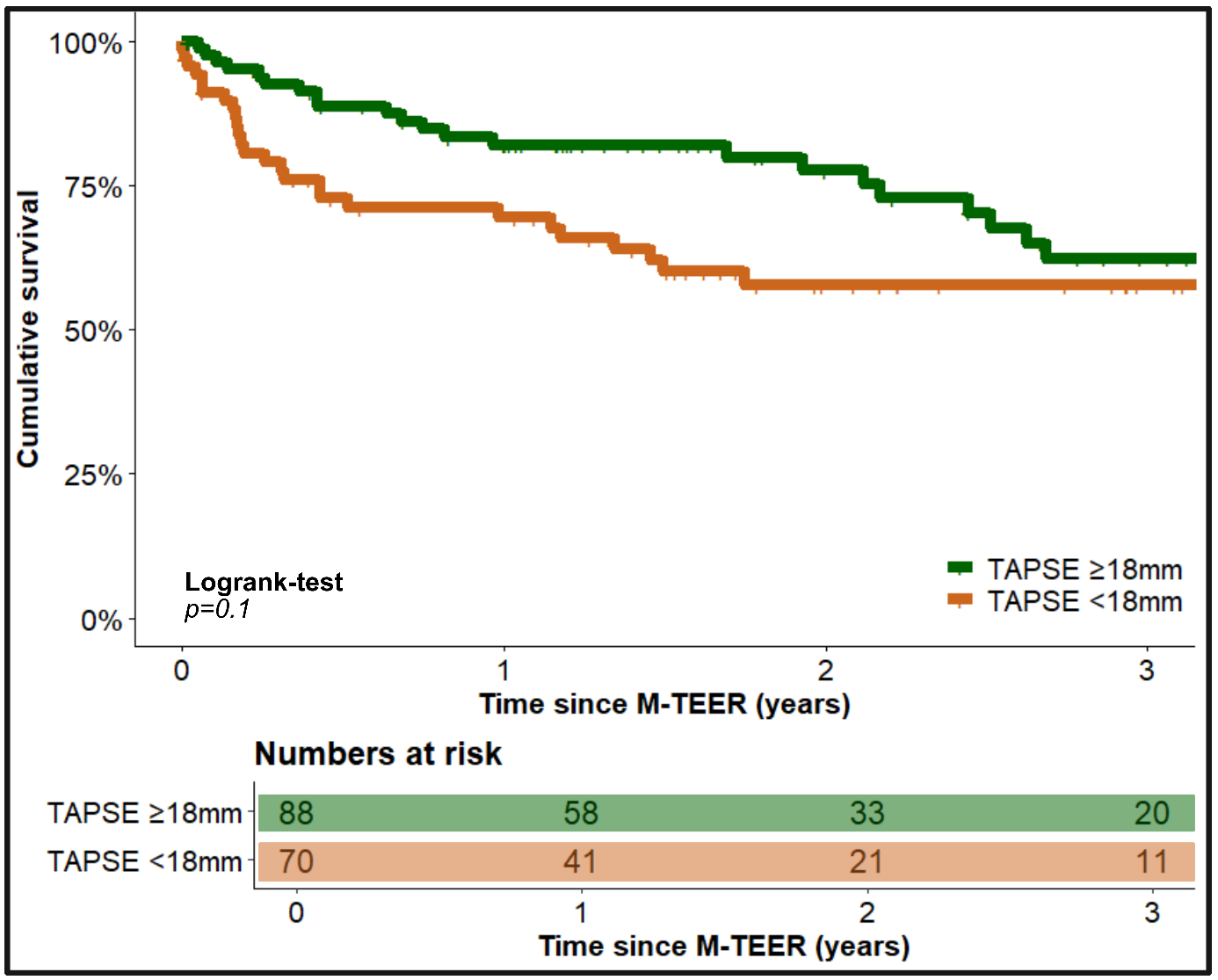
**

**Supplementary Figure 1: Long-term survival between patients with and without right ventricular dysfunction measured by TAPSE after successful M-TEER**

*M-TEER – transcatheter edge-to-edge mitral valve repair. TAPSE – tricuspid annular pulse systolic excursion.*
